# Influence of Environmental Governance on Deforestation in Municipalities of the Brazilian Amazon

**DOI:** 10.1371/journal.pone.0131425

**Published:** 2015-07-24

**Authors:** Lilian Fernandes Oliveira Dias, David Valentim Dias, William Ernest Magnusson

**Affiliations:** CBIO, Instituto Nacional de Pesquisa da Amazônia, Manaus, Brasil; Federal University of Goiás, BRAZIL

## Abstract

It has been argued that measuring governance at scales smaller than global could be an important management tool. However, current studies are conducted on a global scale and use expensive methods. In the present study, we assess whether the reported governance of Amazonian municipalities is related to reductions in deforestation. Economic activity (EA) affected general governance (G) positively (G = 0.81 +1.19 * EA, F1, 98 = 77.36, p < 0.001). Environmental governance (EG) was not affected significantly (p = 0.43) by deforestation before 2000 (PD), but increased significantly (p < 0.001) with general governance (G) (EG = -0.29 + 0.04 PD+0.98*OG, F2,97 = 42.6, p <0.001). Deforestation was not significantly related to environmental governance (p = 0.82). The only indirect effect of significant magnitude was the effect of the density of forest reserves on recent deforestation through deforestation before 2000, which was strongly negative (-0.49). It is possible to assess reported actions to promote municipal governance through official data. However, it is not enough to assume that general governance or environmental governance at the municipal level, as reflected in the official statistics, benefits environmental conservation. In fact, even at the level of nation states, at which most quantification of governance has been undertaken, it seems that the relationship between governance and environmental preservation is only an assumption, because we are aware of no study that supports that hypothesis quantitatively.

## Introduction

The concept of governance has gained international attention during the last decade due to recognition of the need to explore the borders between state and society [1]. The concept of governance is basically that the state is not the only authority that establishes rules, and that dialog among public entities, economic agents, and stakeholders is necessary for the welfare of society as a whole. Governance is the way in which power is exercised in the management of social and economic resources of a country with the aim of development [2, 3]. Governance is a diffuse concept, so that it can be applied to different areas, such as business administration (corporate governance), the application of resources of information technology in public administration and public policy organizations (e-governance), or ways to combat bribery and corruption of public officials (public governance). Environmental governance is the institutional framework of rules, institutions, processes and behavior that affect the way in which powers are exercised in the sphere of political relations or actions related to the ecological system [4]. Goals supported by governance are considered to be more enduring [5], and debate about governance of forests, especially tropical forests, has become intense, both within Brazil and internationally [6].

Most studies of governance are conducted on a global scale, because the complex variables that compose governance are hard to collect, making it difficult to operate on smaller scales [7]. Governance is reflected by many variables, and the World Bank considers hundreds of individual measures in order to evaluate the various dimensions of governance [8]. The indicators are selected to reflect perceptions of governance in the public and private sectors, in non-governmental organizations, as well as the perception of hundreds of citizens and companies, and are quantified through surveys and questionnaires.

To create a database of information coming from many different sources in a reasonable time frame, it is necessary to use techniques of automatic data collection. Public databases can be rich in information [9], but their assessment must be systematic and careful in order to ensure adequate depth and coverage [10]. Although governance is usually compared among nations, measurements on a local scale could be useful management tools, since many problems, such as transparency, corruption potential, lack of equity, and access to technology and media, occur on a local scale [10].

Although deforestation is affected by many factors, such as colonization policies in the past [11, 12], migratory processes and investment in infrastructure [13, 14], logging [15], ranching [16], agrobusiness [17], and previous infrastructure, which produces spatial autocorrelation of deforestation [18, 19] governance is considered an important tool for avoiding deforestation [20]. However, few studies have related quality of governance to deforestation rates. Those that have, focused on scales larger than municipal, but concluded that increase in the quality of governance tends to be associated with a decrease in deforestation rates [21, 22].

It is often claimed that municipal participation is imperative for fighting deforestation. The municipality represents the smallest sphere of government in Brazil, and has relative autonomy in finance, politics, and management. This autonomy, although not representing auto-sufficiency, affects formulation and implementation of public policies [23]. Deforestation in the Amazon reflects the socioeconomic parameters of each municipality [11].

Municipal governments have responsibilities for environmental management, some of which are exclusive and some of which are common to other governmental spheres. Therefore, local official statistics can reflect, albeit indirectly, the governance of the municipality. It is important to distinguish between measures of governance available to decision makers and effective governance. Measures of governance available from official sources (reported governance), such as those used by the World Bank, may not be reflected in effective governance. It would obviously be best to measure effective governance with detailed studies in each political unit (countries in the case of the World Bank studies or municipalities in the case of this study). However, this option is presently too expensive to be used in the development of public policies, especially as effective governance may change from one year to the next. In this study, we evaluate whether reported governance in Amazonian municipalities is related to reduction of forest clearing, which is a major objective of governance in the Brazilian Amazon [24, 25].

## Materials and Methods

### Study site

**Fig 1:**
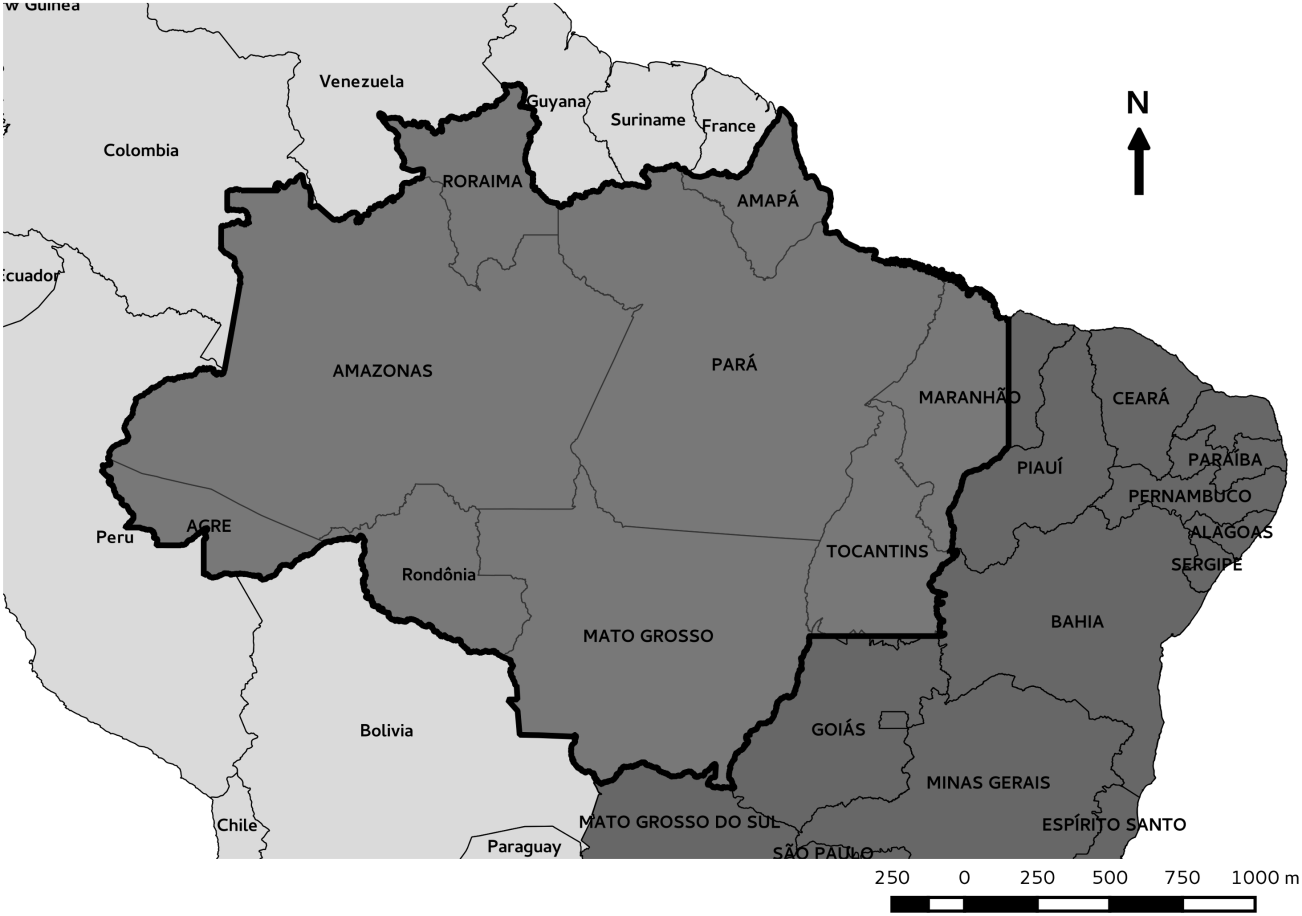
Brazilian Amazon. Brazilian Amazon (large boundary) with yours the state names, Brazil (dark gray) and South America with countrys names (light gray).

The Amazon biome (Fig 1) is present in nine South American countries, with 69% in Brazil [26]. This study included 780 municipalities of the Brazilian Legal Amazon, which comprises the Brazilian States of Amazonas, Roraima, Pará, Amapá, Acre, Rondônia, Mato Grosso, Tocantins, and Maranhão.

**Fig 2:**
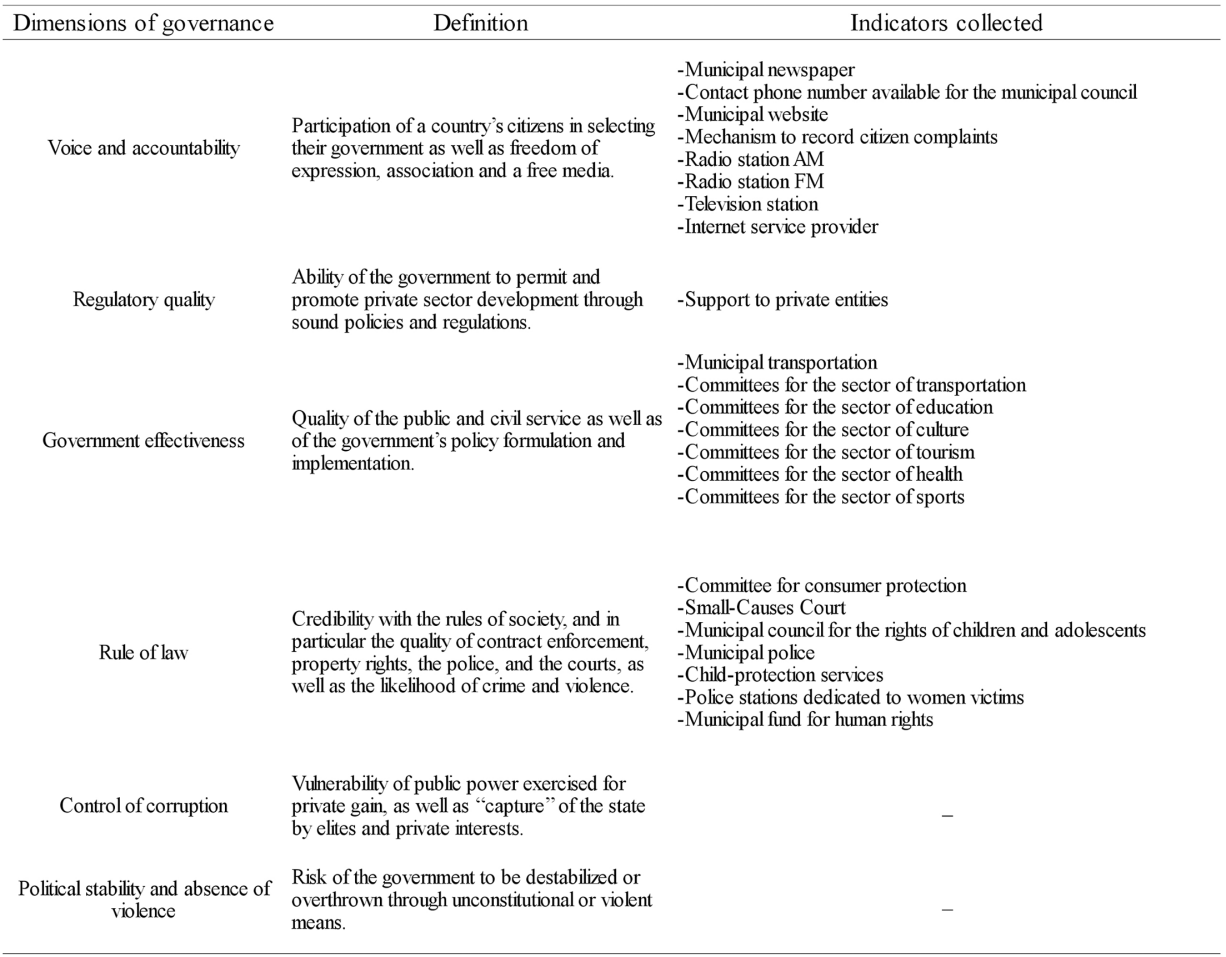
Dimensions of governance and indicators collected. Dimensions of governance established by the World Bank, their definitions and indicators collected from official statistics.

There is no generally accepted method of evaluating governance for municipalities, so we adjusted our methods to reflect those use by the World Bank for evaluating governance in nation states. The World Bank divides governance into six dimensions (Fig 2). In this study, we sought official statistics that reflected as much as possible those dimensions.

Indices of governance not directly related to environmental issues, which hereafter will be referred to as reported general governance, and environmental governance in the municipalities were obtained from the data provided by IBGE (Brazilian Institute of Geography and Statistics) (http://www.ibge.gov.br/home/estatistica/economia/perfilmunic/defaulttab1_perfil.shtm) [27]. The surveys conducted by IBGE covered information regarding social indicators, human rights, municipal management, housing, health, education, sanitation, and environment, among others. In this study, we used data from the 780 municipalities in the Legal Amazon collected between 2001 and 2011.

The presence or absence of those factors was determined using data obtained from the IBGE website [27]. The values of presence (1) or absence (0) were summed in order to obtain the final value for governance (RGG), which potentially ranged from zero to 23.

For environmental governance (REG), we considered only governance indicators related to environmental management. These were organizations or actions that are designed to affect environmental quality or the extent of forest cover. Environmental governance was quantified through presence or absence of the following institutions: environmental council, municipal fund for the environment, availability of resources specifically for the environmental sector, environmental licensing of local impacts, river-basin committee, management of solid waste, management of urban rainwater, municipal council for sanitation, and legislation about selective waste collection.

As for governance, data were obtained from the IBGE website [27]. The values of presence (1) or absence (0) were summed to produce a final value for environmental governance, which potentially ranged between zero and 9.

The World Bank uses a system of questionnaires to evaluate expert opinion on the relative importance of each indicator and weights individual indicators accordingly. This system is subjective and difficult to reproduce for municipalities. Therefore, we used a Bayesian hierarchical analysis to attribute weights to individual indicators to maximize their relationship to deforestation. This allowed us to evaluate whether a weighting system would change our conclusions.

GDP (Gross Domestic Product) is the most commonly used indicator to measure economic activity. It reflects wealth production in a location, and indicates the capacity of the economy to generate jobs [28]. The economic activity (EA) index consisted of the GDP annual value for each municipality, obtained from IBGE website [29]. Other indicators, such as the Human Development Index (HDI), that include historical factors and economic effects confound the results of effective governance.

The areas that were deforested (PD and D) in each municipality were obtained from the PRODES (Program to calculate deforestation in the Amazon) database, in the INPE (National Institute for Space Research) website [30]. More details about the deforestation of the sampling method in www.obt.inpe.br/prodes/metodologia_TaxaProdes.pdf.

The areas covered by state and federal reserves (FRD) were obtained from georeferenced vector layers of conservation units and Brazilian municipalities available in the MMA (Ministry of Environment) website [31]. The extent of official state and federal roads (RD) was obtained from georeferenced vector layers of highways and towns in Brazil available in the DNIT (National Department of Infrastructure and Transportation) website [32]. The values for roads and reserves were transformed into density by dividing the total area of the municipality by the area occupied by reserves and total length of roads.

The factors that affect deforestation are spatially correlated. Deforestation usually occurs in scattered patches, such that municipalities included in the same patch have similar levels of deforestation. Therefore, the information from municipalities close to each other is often not independent, and such lack of independence compromises statistical analyses [33].

**Fig 3:**
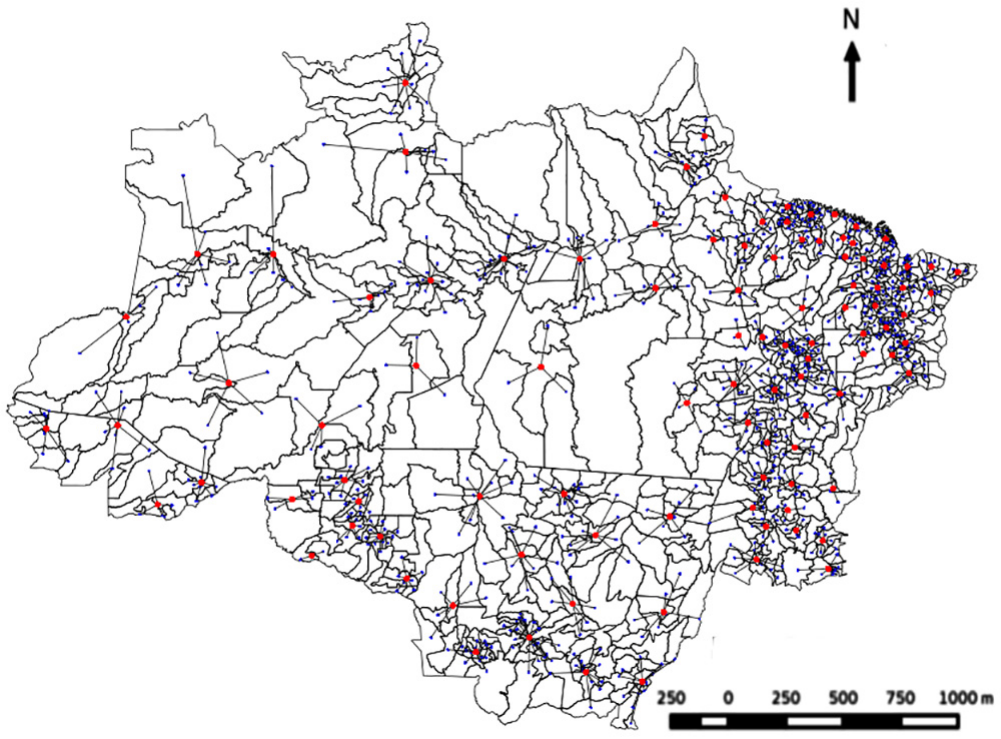
Supermunicipalities of the Brazilian Amazon. Sites for municipalities (blue dots), and supermunicipalities (red dots) formed after grouping.

In order to minimize this problem, spatially close municipalities with similar deforestation were clustered using the K-means clustering algorithm. The clustering parameters were latitude and longitude of the municipal headquarters and deforestation. The municipalities were clustered into 100 groups (hereafter supermunicipalities), which was the number considered to be the minimum to maintain confidence in the statistical analyses (Fig 3).

Simple regression coefficients among variables do not take into account the direct and indirect effects of predictor variables. Path analysis was used to quantify indirect effects. This procedure estimates the magnitude of the effects of predictor variables on comparable scales through standardized regression coefficients (PC) and allows the assessment of effects of one variable that propagate through intermediate effects of other variables.

## Results

**Fig 4:**
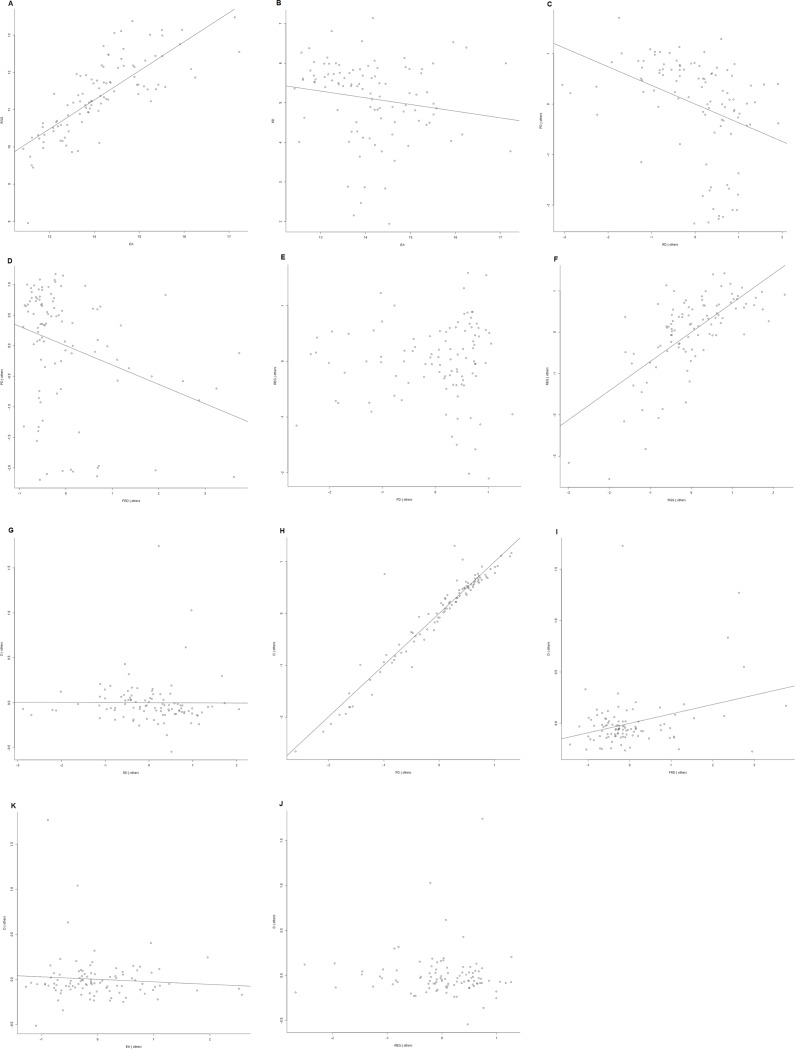
Panel results graphs. Panel with graphs of the simple regression and partial graphs of the multiple regression.

Economic activity (EA) affected reported governance not directly related to environmental issues, which hereafter will be referred to as reported general governance (RGG), positively (RGG = 0.81 + 1.19 * EA, F1, 98 = 77.36, p < 0.001) (Fig 4a), and road density (RD) was significantly related (p = 0.01) to economic activity (RD = 0.6–0.6 * EA, F1, 98 = 6.4, p = 0.01) (Fig 4b). Road density (p < 0.001) and forest-reserve density (FRD) (p = 0.0002) had negative relationships with deforestation before 2000 (PD): (PD = 1.02–0.74 * RD -0.54 * FRD, F2,97 = 14.0, p < 0.001) (Fig 4c) (Fig 4d).

Reported environmental governance (REG) was not significantly affected (p = 0.43) by deforestation before 2000 (PD) (Fig 4e), but increased significantly (p < 0.001) with the reported general governance (RGG) (Fig 4f): (REG = -0.29 + 0.04 * PD + 0.98 * RGG, F2,97 = 42.6, p < 0.001).

Deforestation (D) was related significantly (p = 0.004) and negatively with road density (RD) (Fig 4g), significantly (p < 0.001) and positively with deforestation before 2000 (PD) (Fig 4h), significantly (p = 0.007) and positively with forest reserve density (FRD) (Fig 4i), but was not significantly related to reported environmental governance (REG) (p = 0.82) (Fig 4j) or economic activity (EA) (p = 0.32) (Fig 4k): (D = 0.08–0.13 * RD + 0.91 * PD + 0.09 * FRD + 0.01 * REG– 0.11 * EA, F5,94 = 400.4, p < 0.001).

**Fig 5:**
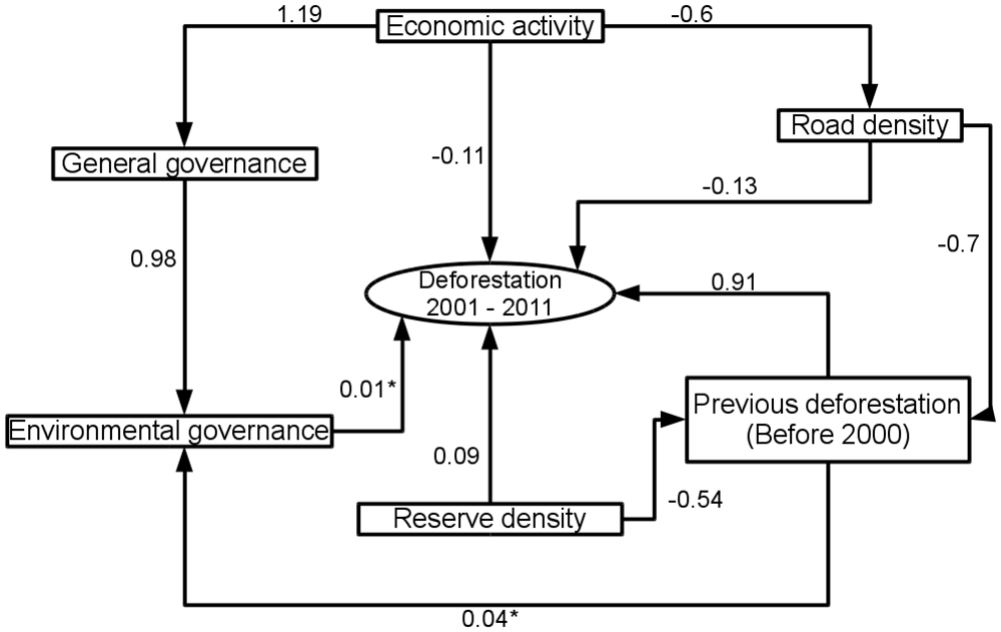
Path analysis results. Each arrow represents a path and its associated path coefficient. Asterisks represent statistically insignificant relationships in simple or multiple regression tests (P > 0.05).

Path analysis indicated that indirect effects on deforestation between 2001 and 2010 were generally very low in comparison with the direct effects, and that most indirect effects had path coefficients (PC) lower than 0.1 (Fig 5). Economic activity had indirect positive effects on deforestation through general governance and environmental governance (path coefficient 0.01). It also had a slightly higher indirect positive effect through the effect of road density on deforestation (0.07). Nevertheless, the indirect effect of road density on current deforestation through deforestation (before 2000) was positive (0.08). Road density had a minor indirect negative effect through deforestation (before 2000) and environmental governance (-0.003). General governance had a positive effect on deforestation through environmental governance (0.011). The indirect effect of deforestation (before 2000) on deforestation through environmental governance on deforestation was positive, but very low (0.0004). The indirect effect of forest reserve density through deforestation before 2000 and environmental governance was also very low (-0.0002).

Overall, the only indirect effect of significant magnitude was the effect of forest-reserve density on recent deforestation through deforestation before 2000, which was strongly negative (-0.49).

## Discussion

Governmental institutions have much information available to use in both internal operations and provision of services [34]. Nevertheless, data on general governance and environmental governance in municipalities were inconsistently published, reducing the quality, and complicating access to the information. The survey forms used by IBGE varied among years, so in this study it was necessary to develop automated data-search methods on the internet to obtain information within a reasonable time frame. Despite the difficulty of access, indicators that reflect the quality of municipal public management are essential for effective planning, since they allow monitoring of economic and social development in the municipalities [35]. Also, it is only possible to assess the effectiveness of governance actions if they can be quantified.

The strong positive relationship between economic activity and reported governance was similar to the relationship reported in the literature for units larger than municipalities and for models derived from cellular automata. This is most likely due to the fact that most political decisions made in Brazil target strong economic growth [36, 37]. Municipalities that are more urbanized and more economically developed usually have more environmental problems, even though they have more governmental institutions responsible for the environment [38]. In the Legal Amazon, economic activity is strongly linked to land use [39], which results in public policies to regulate it [40].

The relationship between reported governance and reported environmental governance also behaved similarly to what is reported in the literature and in models of cellular automata in units larger than the municipality. Maintaining a system of economic progression demands environmental policies that make it possible. This system tends to reduce the ability of the sectors responsible for environmental issues to influence public policies [41].

The road network is responsible for most of the outflow of Amazonian products [42]. Roads could enable economic activities with negative environmental impact. The roads can give access to migrants and entrepreneurs with different levels of economic resources. This increases the value of the land, stimulating real-estate speculation and, consequently, expansion of deforestation [43]. However, at the municipal level, economic activity had a weak negative effect on roads, possibly because data collected on economic activity do not reflect the profit obtained from illegal activities conducted in municipalities of the Legal Amazon, or because we evaluated only official roads.

Deforestation in the Amazon is associated with road construction [44]. Nevertheless, one model of deforestation indicated that, if the construction of roads was made within a scenario of effective governance, deforestation could be reduced by 62% for the Brazilian Legal Amazon, and 55% for the basin as whole [20]. At the municipal level, we did not find a relationship between road density and deforestation between 2000 and 2010. There was also a negative relationship between road density and deforestation (before 2000). Data collected about the road network included only state and federal roads, and did not quantify informal roads, which might have contributed to underestimation of extent of the road network in the Legal Amazon and, consequently, the deforestation caused by it. More studies about these relationships are needed, since the roads that are planned by public authorities are potentially part of governance, and may have little effect on deforestation compared to roads associated with the informal economy.

There was a negative relationship between the density of forest reserves and the deforestation that occurred up to the year 2000, as the implementation of reserves limits the area to be deforested [45]. However, the lack of infrastructure needed for reserve operation (fiscal agents, cars, access ways, etc.) added to an inefficient justice system and to market incentives for continuing exploitation, can make this relationship weak and positive in the long term. This was observed in the relationship between forest reserve density and deforestation in municipalities of the Legal Amazon between the years of 2001 and 2011 in this study, and also the studies by Machado *et al*. [46], Azevedo & Saito [47] and Almeida *et al*. [48].

The indices used here to describe governance and environmental governance had no significant effect on deforestation. It is possible that official data do not effectively reflect governance. However, the assessment of governance at higher levels, such as among nation states, is made through official data [49], and the verification in loco of 780 municipalities would be economically impracticable.

The absence of a strong effect of reported governance on deforestation possibly results from the fact that the main activities causing deforestation in the Legal Amazon are associated with illegal activities, which are often difficult to detect with data obtained from official sources. The assessment of governance has usually been made at the level of countries, in which the institutions that propagate governance activities are distant from the activities that governance should repress. Local residents and their representatives in municipalities affected by deforestation may oppose creation of reserves or the implementation of restrictive environment policies. The absence of effective environmental policies may bring immediate benefits (jobs in agricultural and extractive industries, fisheries, etc.), and these benefits are likely to be more important for local people than concerns about deforestation [50, 51, 52].

It is generally assumed that degradation of the environment is a function of governance. However, it is just as likely that the relationship is the inverse. When there is little environmental degradation, there is little pressure on government agencies to implement environmental governance. Degradation of environmental conditions leads to demands on local government to implement governance actions that will be reflected in official statistics. Therefore, it may be that governance actions generally come too late to avoid environmental degradation, such as deforestation.

It is possible to assess actions to promote municipal governance through official data, and reported governance may have effects on environmental concerns other than deforestation. However, it is not enough to assume that governance or environmental governance at the municipal level will benefit environment conservation, and studies must be undertaken to evaluate the relationship between governance and every environmental aspect that governance is supposed to improve. In fact, even at the level of nation states, at which most quantification of governance has been undertaken, it seems that the relationship between governance and environment preservation is only an assumption, because we are aware of no studies that support that hypothesis quantitatively. It may be that reported governance reflects more attempts by people to recover environmental quality that they have lost, rather than a mechanism to avoid environmental degradation.

## Supporting Information

S1 File Additional information. Supporting information with data table, statistical analyzes, document explaining the governance indicators and a list of software and packges used.
(ZIP)
