# Supplementary material for: Influence of Environmental Governance on Deforestation in Municipalities of the Brazilian Amazon
Source: PLoS One. 2015 Jul 24;10(7):e0131425. doi: 10.1371/journal.pone.0131425 (PMC4514646; doi:10.1371/journal.pone.0131425)
Supplement: S1 File — Supporting information with data table, statistical analyzes, document explaining the governance indicators and a list of software and packges used. (ZIP) [file pone.0131425.s001.zip › support_information/model_desmatamento.pdf]

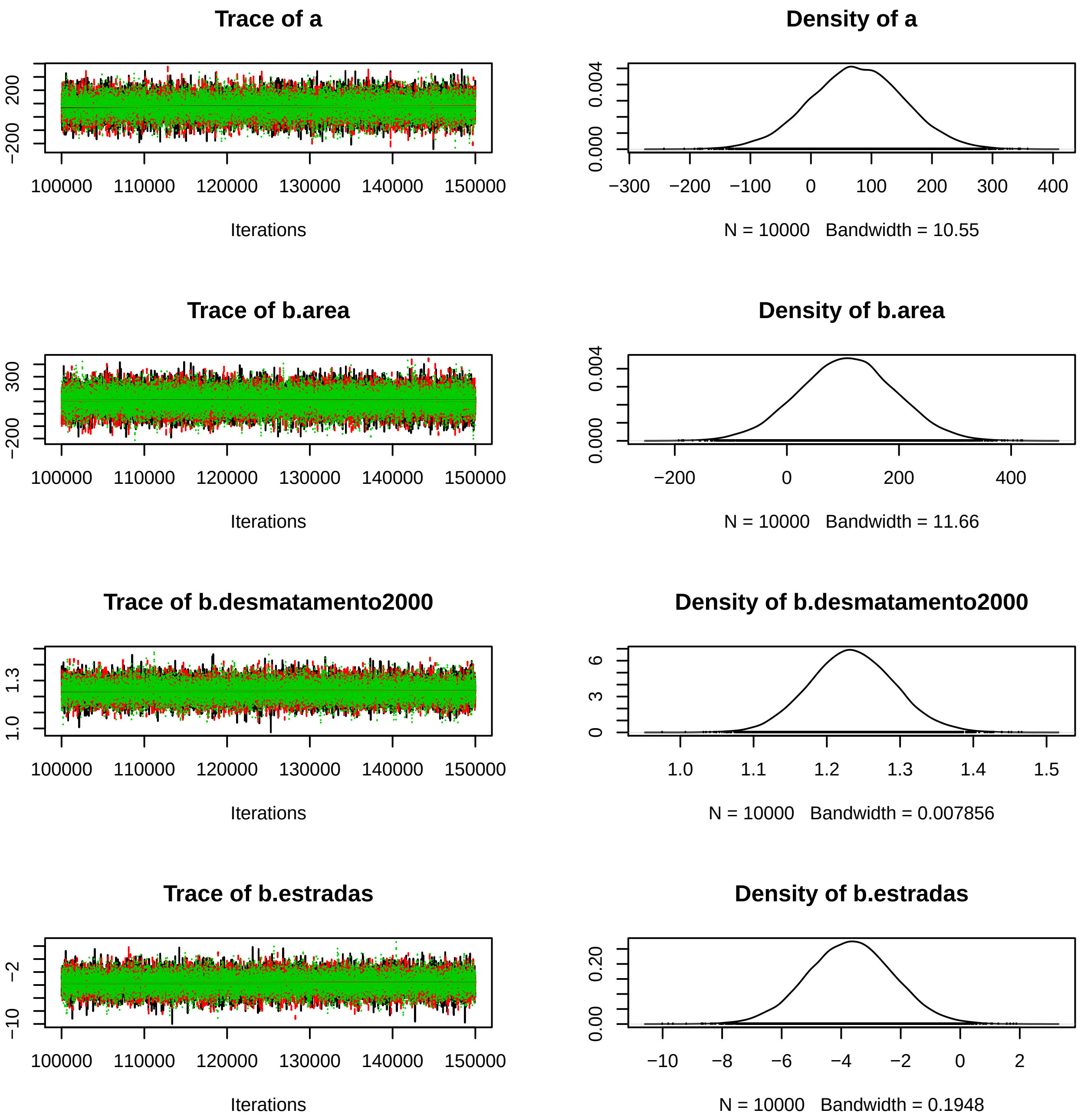
Trace of a
200
−200
100000 110000 120000 130000 140000 150000
Iterations
Density of a
0.000 0.004
−300 −200 −100 0 100 200 300 400
N = 10000 Bandwidth = 10.55
Trace of b.area
300
−200
100000 110000 120000 130000 140000 150000
Iterations
Density of b.area
0.000 0.004
−200 0 200 400
N = 10000 Bandwidth = 11.66
Trace of b.desmatamento2000
1.0 1.3
100000 110000 120000 130000 140000 150000
Iterations
Density of b.desmatamento2000
0 3 6
1.0 1.1 1.2 1.3 1.4 1.5
N = 10000 Bandwidth = 0.007856
Trace of b.estradas
−10 −2
100000 110000 120000 130000 140000 150000
Iterations
Density of b.estradas
0.00 0.20
−10 −8 −6 −4 −2 0 2
N = 10000 Bandwidth = 0.1948

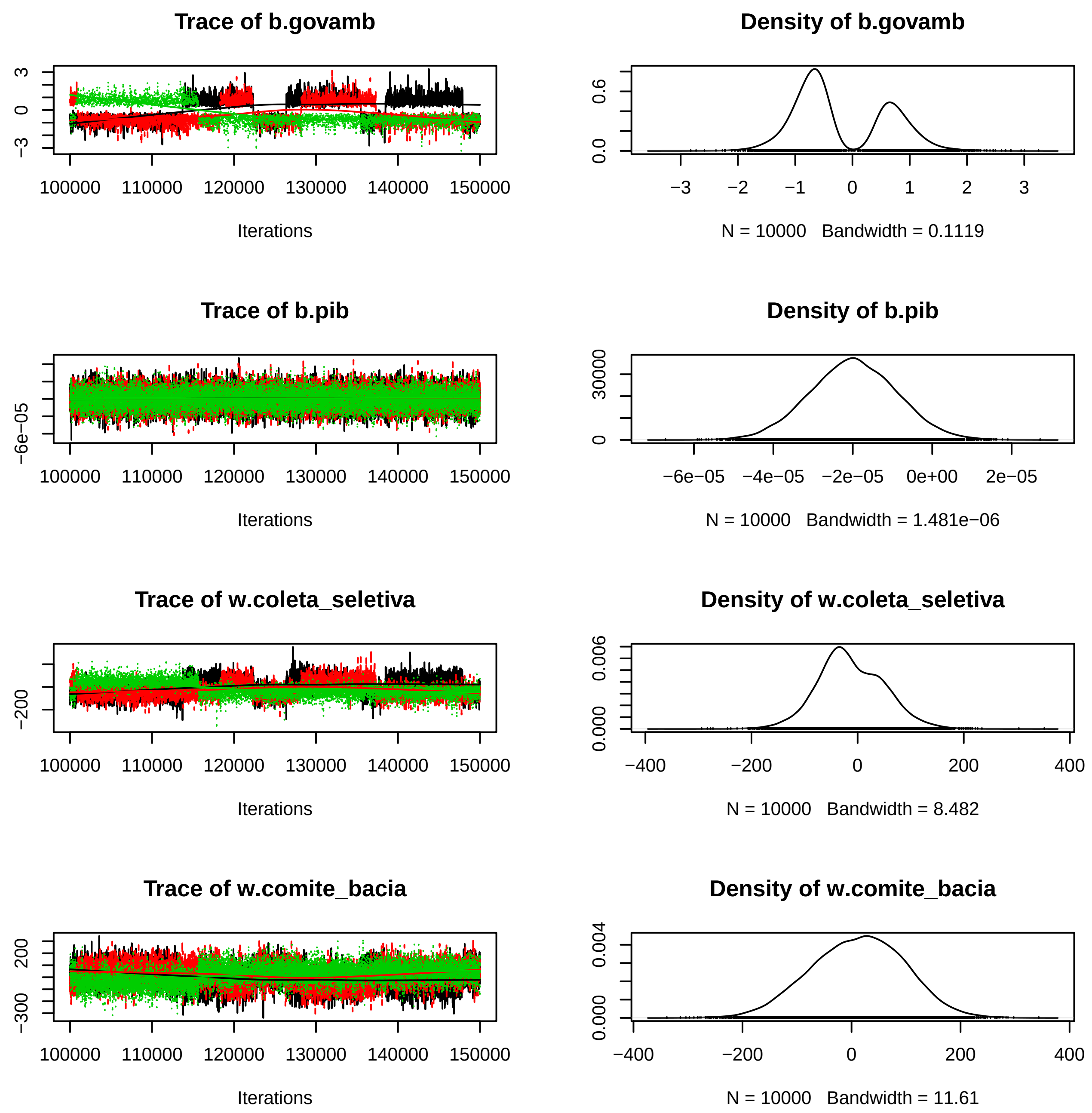

Trace of b.govamb
Density of b.govamb
Iterations
N = 10000 Bandwidth = 0.1119
Trace of b.pib
Density of b.pib
Iterations
N = 10000 Bandwidth = 1.481e−06
Trace of w.coleta_seletiva
Density of w.coleta_seletiva
Iterations
N = 10000 Bandwidth = 8.482
Trace of w.comite_bacia
Density of w.comite_bacia
Iterations
N = 10000 Bandwidth = 11.61

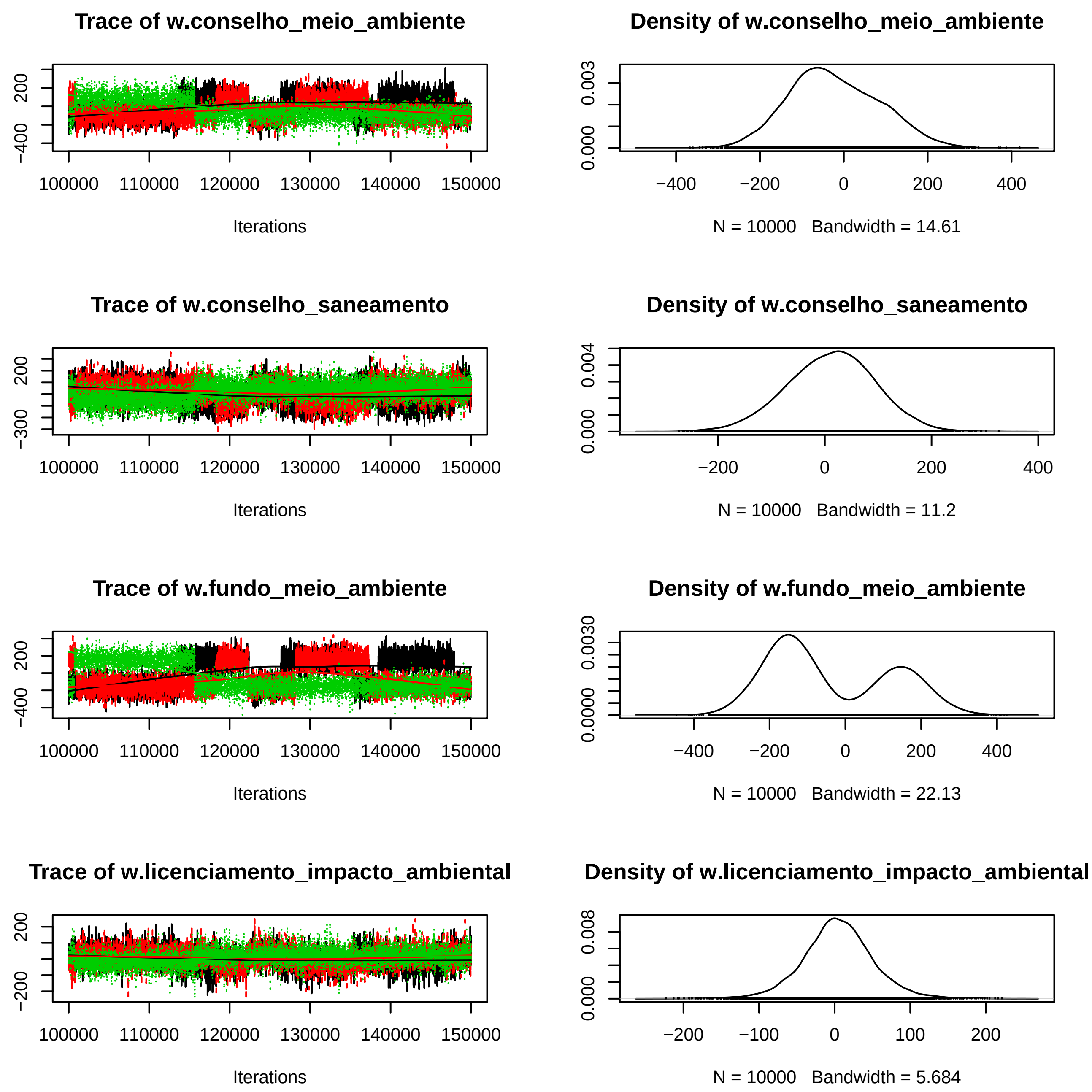
Trace of w.conselho_meio_ambiente
200
−400
100000 110000 120000 130000 140000 150000
Iterations
Density of w.conselho_meio_ambiente
0.000 0.003
−400 −200 0 200 400
N = 10000 Bandwidth = 14.61
Trace of w.conselho_saneamento
200
−300
100000 110000 120000 130000 140000 150000
Iterations
Density of w.conselho_saneamento
0.000 0.004
−200 0 200 400
N = 10000 Bandwidth = 11.2
Trace of w.fundo_meio_ambiente
200
−400
100000 110000 120000 130000 140000 150000
Iterations
Density of w.fundo_meio_ambiente
0.0000 0.0030
−400 −200 0 200 400
N = 10000 Bandwidth = 22.13
Trace of w.licenciamento_impacto_ambiental
200
−200
100000 110000 120000 130000 140000 150000
Iterations
Density of w.licenciamento_impacto_ambiental
0.000 0.008
−200 −100 0 100 200
N = 10000 Bandwidth = 5.684

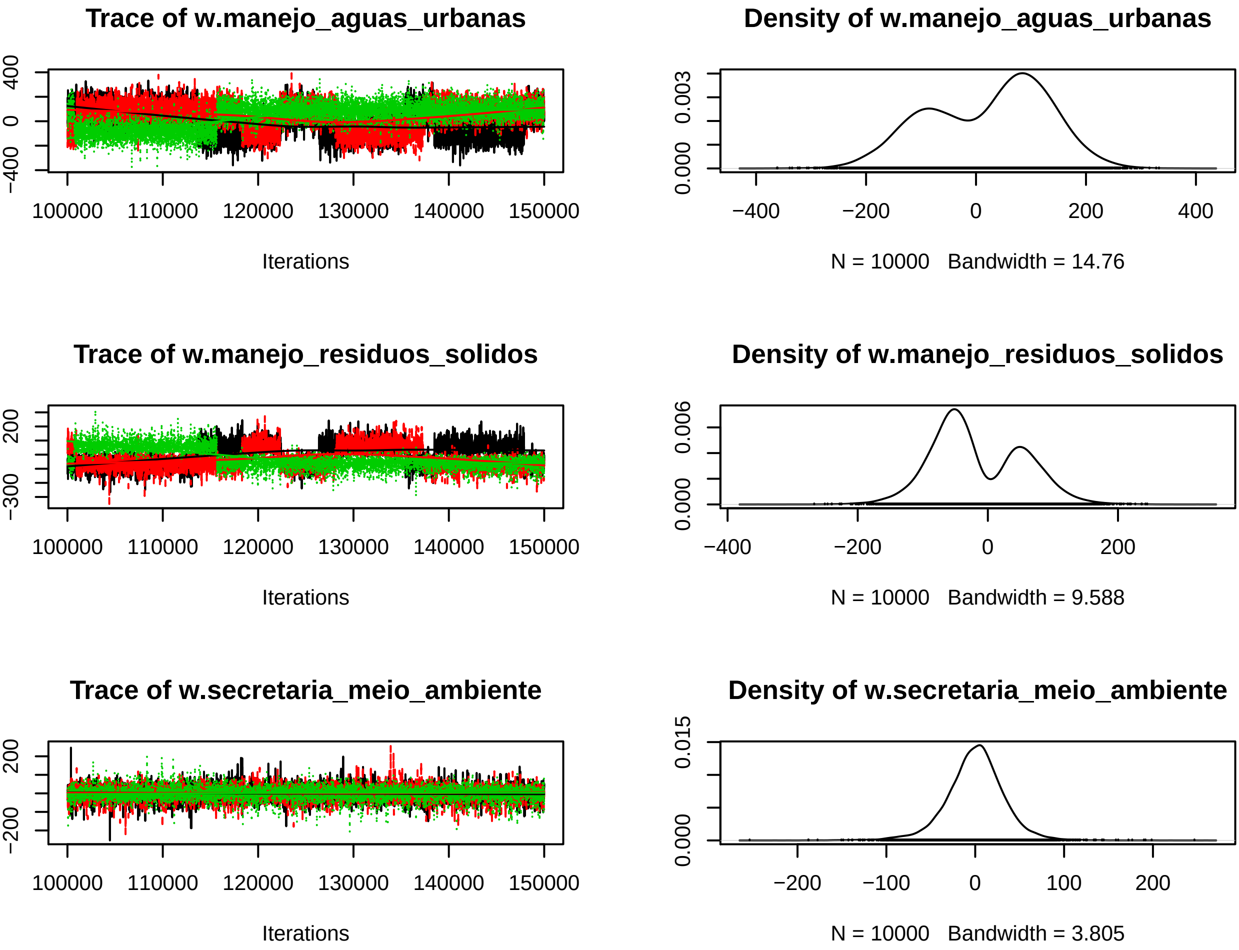
Trace of w.manejo_aguas_urbanas
Density of w.manejo_aguas_urbanas
−400 0 400
100000 110000 120000 130000 140000 150000
Iterations
0.000 0.003
−400 −200 0 200 400
N = 10000 Bandwidth = 14.76
Trace of w.manejo_residuos_solidos
Density of w.manejo_residuos_solidos
−300 200
100000 110000 120000 130000 140000 150000
Iterations
0.000 0.006
−400 −200 0 200
N = 10000 Bandwidth = 9.588
Trace of w.secretaria_meio_ambiente
Density of w.secretaria_meio_ambiente
−200 200
100000 110000 120000 130000 140000 150000
Iterations
0.000 0.015
−200 −100 0 100 200
N = 10000 Bandwidth = 3.805
