# Supplementary material for: Influence of Environmental Governance on Deforestation in Municipalities of the Brazilian Amazon
Source: PLoS One. 2015 Jul 24;10(7):e0131425. doi: 10.1371/journal.pone.0131425 (PMC4514646; doi:10.1371/journal.pone.0131425)
Supplement: S1 File — Supporting information with data table, statistical analyzes, document explaining the governance indicators and a list of software and packges used. (ZIP) [file pone.0131425.s001.zip › support_information/model_desmatamento2000.pdf]

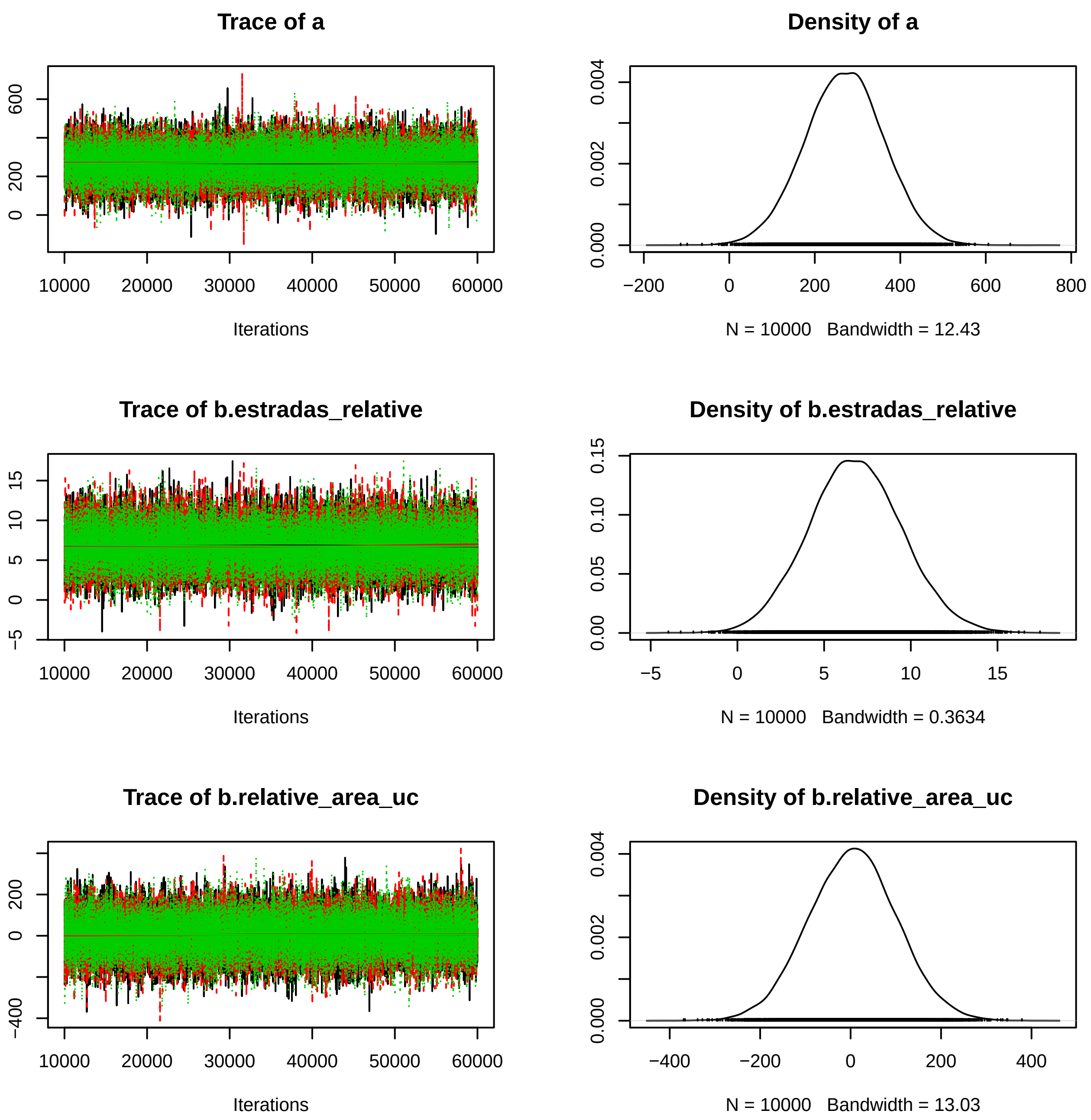

Trace of a
600
200
0
10000
20000
30000
40000
50000
60000
Iterations
Density of a
0.004
0.002
0.000
−200
0
200
400
600
800
N = 10000 Bandwidth = 12.43
Trace of b.estradas_relative
15
10
5
0
−5
10000
20000
30000
40000
50000
60000
Iterations
Density of b.estradas_relative
0.15
0.10
0.05
0.00
−5
0
5
10
15
N = 10000 Bandwidth = 0.3634
Trace of b.relative_area_uc
200
0
−400
10000
20000
30000
40000
50000
60000
Iterations
Density of b.relative_area_uc
0.004
0.002
0.000
−400
−200
0
200
400
N = 10000 Bandwidth = 13.03
