# Supplementary material for: Influence of Environmental Governance on Deforestation in Municipalities of the Brazilian Amazon
Source: PLoS One. 2015 Jul 24;10(7):e0131425. doi: 10.1371/journal.pone.0131425 (PMC4514646; doi:10.1371/journal.pone.0131425)
Supplement: S1 File — Supporting information with data table, statistical analyzes, document explaining the governance indicators and a list of software and packges used. (ZIP) [file pone.0131425.s001.zip › support_information/supportinformation1.docx]

GOVERNANCE INDICATORS

The choice of indicators to measure governance was based on the definitions of dimensions of governance given by the World Bank. The indicators listed here seek to extract the maximum of the essence of each of the proposed dimensions for definition of governance, within the availability of data from the database used in this study.

**Voice and accountability**

This dimension considers freedom of expression associated with free media, and the ability of citizens of a country to participate in the choice of government. Voice and accountability in the present study considered indicators that reflect the variability of resources that promote freedom of expression (Television station, Radio station AM, Radio station FM, Internet service provider), as well as tools where citizens can obtain information and express opinions about the government (Municipal newspaper, Contact phone number available for the municipal council, Municipal website, Mechanism to record citizen complaints).

**Regulatory quality**

Regulatory quality seeks to measure the government's ability to provide policies and regulations that enable private-sector development. For the present study, we quantified this by the presence or absence of support to private entities.

**Government effectiveness**

Government effectiveness aimed to quantify government actions aimed at increasing the quality of public services, competence of public administration and political independence, as well as the quality of policy formulation. To measure attempts to increase government effectiveness, we sought information on the existence of public services (municipal transportation), and information indicating the existence of a dialogue between citizens and the government of their respective municipality (Committees for the Transportation sector, Committees for Education, Committees for Culture, Committees for Tourism, Committees for Health, Committees for Sport). It is important to note that we were interested in the effective of governance actions on effective governance (reduction in deforestation). Therefore, our statistics reflect attempts at effective governance rather than the effect of governance per se.

**Rule of Law**

This dimension aims at identifying how much agents trust the rules of society and they act in accordance with them, including the quality of contract enforcement and property rights, the police and the courts, as well as the likelihood of crime and violence. For this dimension, we sought indicators providing information about the existence of entities that would ensure protection by the law for citizens in general (Committee forC Protection, Small-Causes Court, Municipal Ppolice) as well as citizens included in possible risk groups (Municipal Council for the Rights of Children and Adolescents, Child-Protection Services, Police stations dedicated to women victims, Municipal fund for human rights). All these actions promote the rule of law, but do not necessarily guarantee it.

**REFERENCES**

World Bank. (1992) Governance and development. Washington, D.C; World Bank.

World Bank (2014) Worldwide Governance indicators. http://info.worldbank.org/governance/wgi/index.aspx#home. Washington. D.C; World Bank.

World Bank. (2008). Governance Matters. Indicadores de governança 1996-2007. Washington. D.C. World bank.
