# Supplementary material for: Influence of Environmental Governance on Deforestation in Municipalities of the Brazilian Amazon
Source: PLoS One. 2015 Jul 24;10(7):e0131425. doi: 10.1371/journal.pone.0131425 (PMC4514646; doi:10.1371/journal.pone.0131425)
Supplement: S1 File — Supporting information with data table, statistical analyzes, document explaining the governance indicators and a list of software and packges used. (ZIP) [file pone.0131425.s001.zip › support_information/supportinformation3.docx]

Software and packges

R Core Team (2014). R: A language and

environment for statistical computing. R

Foundation for Statistical Computing,

Vienna, Austria. URL

http://www.R-project.org/.

Martyn Plummer, Nicky Best, Kate Cowles

and Karen Vines (2006). CODA: Convergence

Diagnosis and Output Analysis for MCMC, R

News, vol 6, 7-11

R Special Interest Group on Databases

(2014). DBI: R Database Interface. R

package version 0.3.0.

http://CRAN.R-project.org/package=DBI

Chris Brunsdon and Hongyan Chen

(2014). GISTools: Some further

GIS capabilities for R. R package

version 0.7-3.

http://CRAN.R-project.org/package=GISTools

G. Grothendieck (2014). gsubfn:

Utilities for strings and

function arguments.. R package

version 0.6-6.

http://CRAN.R-project.org/package=gsubfn

Original S code by Richard A.

Becker and Allan R. Wilks. R

version by Ray Brownrigg.

Enhancements by Thomas P Minka

<tpminka@media.mit.edu> (2014).

maps: Draw Geographical Maps. R

package version 2.3-7.

http://CRAN.R-project.org/package=maps

Roger Bivand and Nicholas

Lewin-Koh (2014). maptools: Tools

for reading and handling spatial

objects. R package version

0.8-30.

http://CRAN.R-project.org/package=maptools

Louis Kates and Thomas Petzoldt

(2012). proto: Prototype

object-based programming. R

package version 0.3-10.

http://CRAN.R-project.org/package=proto

Erich Neuwirth (2011).

RColorBrewer: ColorBrewer

palettes. R package version

1.0-5.

http://CRAN.R-project.org/package=RColorBrewer

Roger Bivand, Tim Keitt and Barry

Rowlingson (2014). rgdal:

Bindings for the Geospatial Data

Abstraction Library. R package

version 0.8-16.

http://CRAN.R-project.org/package=rgdal

Roger Bivand and Colin Rundel

(2014). rgeos: Interface to

Geometry Engine - Open Source

(GEOS). R package version 0.3-6.

http://CRAN.R-project.org/package=rgeos

Martyn Plummer (2014). rjags:

Bayesian graphical models using

MCMC. R package version 3-13.

http://CRAN.R-project.org/package=rjags

David A. James, Seth Falcon and

the authors of SQLite (2013).

RSQLite: SQLite interface for R.

R package version 0.11.4.

http://CRAN.R-project.org/package=RSQLite

Seth Falcon (2010). RSQLite.extfuns: Math and String Extension Functions for

RSQLite. R package version 0.0.1.

http://CRAN.R-project.org/package=RSQLite.extfuns

Pebesma, E.J., R.S. Bivand, 2005. Classes and methods for spatial data in R.

R News 5 (2), http://cran.r-project.org/doc/Rnews/.

G. Grothendieck (2014). sqldf: Perform SQL Selects on R Data Frames. R

package version 0.4-7.1. http://CRAN.R-project.org/package=sqldf
