# Supplementary figures and images for: Influence of Environmental Governance on Deforestation in Municipalities of the Brazilian Amazon
Source: PLoS One. 2015 Jul 24;10(7):e0131425. doi: 10.1371/journal.pone.0131425 (PMC4514646; doi:10.1371/journal.pone.0131425)

**Trace of a**

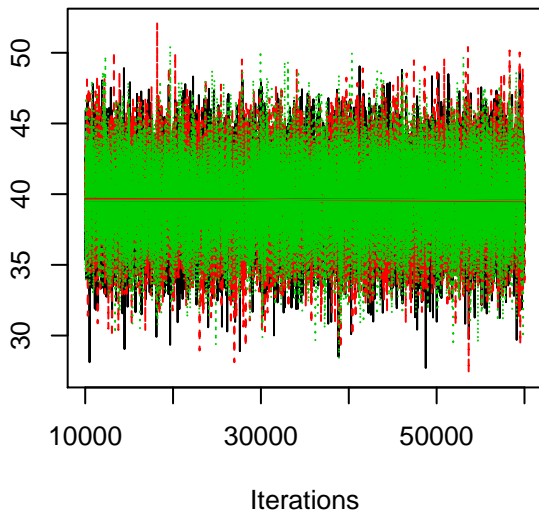

**Density of a**

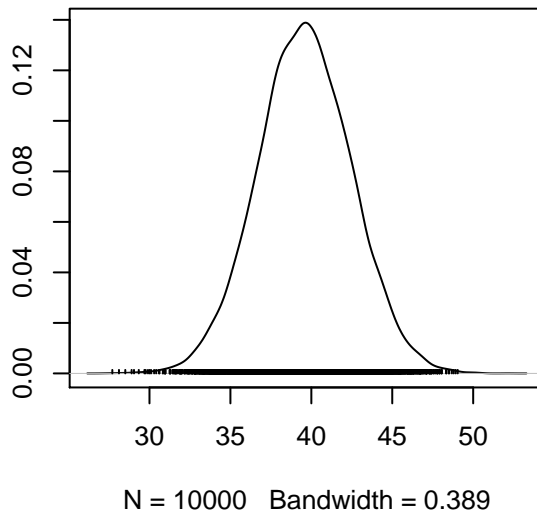

**Trace of b.pib**

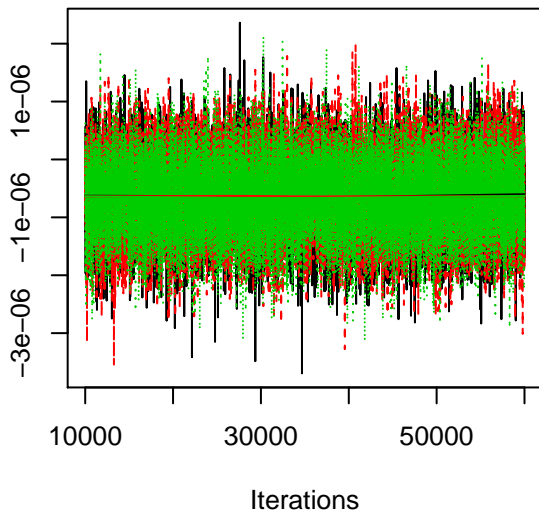

**Density of b.pib**

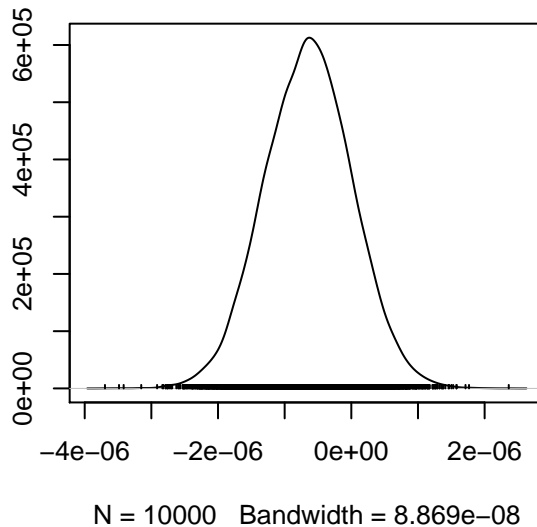

Supplement: S1 File — Supporting information with data table, statistical analyzes, document explaining the governance indicators and a list of software and packges used. (ZIP) [file pone.0131425.s001.zip › support_information/model_estradas_relative.pdf]
